# Dr. Frank P. Foster

**Published:** 1911-09

**Authors:** 


Dr. Frank P. Foster, editor of the *New York Medical Journal* since January 1, 1880, died at Chadwick, N. J., August 13, 1911. He was born at Concord, N. H., November 26, 1841, of old Yankee stock. Educated in the public schools and under the preceptorship of Dr. Lyman Gage, he graduated in medicine from the College of Physicians and Surgeons in 1862. After preliminary practice as interne at the New York Hospital, as ship surgeon on the Pacific, and as contract surgeon in the United States Army during the last years of the Civil War, he engaged in practice in New York City in 1865, at first as a general practitioner, then as a dermatologist but eventually as an obstetrician and gynecologist. He was noted in various ways. Perhaps his most practical service was in introducing the manufacture of bovine vaccine into America in 1860. An author of high literary merit, his greatest service in medical writing, was, after all, his able editorial management of the *New York Medical Journal.* His own journal contains in the issue of August 26, 1911, one of his latest papers, "Forty-six Years of Medicine in New York." It would have been appropriate that this article should be of the nature of an autobiography, as its name suggests. It is characteristic of the man, however, that the author scarcely speaks of himself but gives an interesting review of certain phases of medical history of the city and the state.

We do not remember ever to have met Dr. Foster personally but frequently heard from him in regard to original and occasional editorial contributions to the *New York Medical Journal* and even in the brief correspondence, he injected so much of the spirit of friendship and personal interest, that his death comes to us as a personal loss. Few men have done so much as Dr. Foster in influencing and advising the medical profession for good.

